# Discordant Amyloid Status Diagnosis in Alzheimer’s Disease

**DOI:** 10.3390/biomedicines10112880

**Published:** 2022-11-10

**Authors:** Lorena García-Vallés, Carmen Peña-Bautista, Lourdes Álvarez-Sánchez, Inés Ferrer-Cairols, Miguel Baquero, Consuelo Cháfer-Pericás

**Affiliations:** 1 Alzheimer’s Disease Research Group, Health Research Institute La Fe, Avda de Fernando Abril Martorell, 106, 46026 Valencia, Spain; 2 Division of Neurology, University and Polytechnic Hospital La Fe, 46026 Valencia, Spain

**Keywords:** cerebrospinal fluid sample, amyloid PET, Alzheimer’s disease, diagnosis, neuropsychology

## Abstract

Introduction: Early and accurate Alzheimer’s disease (AD) diagnosis has evolved in recent years by the use of specific methods for detecting its histopathological features in concrete cases. Currently, biomarkers in cerebrospinal fluid (CSF) and imaging techniques (amyloid PET) are the most used specific methods. However, some results between both methods are discrepant. Therefore, an evaluation of these discrepant cases is required. Objective: The aim of this work is to analyze the characteristics of cases showing discrepancies between methods for detecting amyloid pathology. Methodology: Patients from the Neurology Department of La Fe Hospital (n = 82) were diagnosed using both methods (CSF biomarkers and amyloid-PET). Statistical analyses were performed using logistic regression, and sex and age were included as covariables. Additionally, results of standard neuropsychological evaluations were taken into account in our analyses. Results: The comparison between CSF biomarker (Aβ42) and amyloid PET results showed that around 18% of cases were discrepant—mainly CFS-negative and PET-positive cases had CSF levels close to the cut-off point. In addition, a correlation between the episodic memory test and CSF biomarkers levels was observed. However, the same results were not obtained for other neuropsychological domains. In general, CSF- and PET-discrepant cases showed altered episodic memory in around 66% of cases, while 33% showed normal performance. Conclusions: In common clinical practice at tertiary memory centers, result discrepancies between tests of amyloid status are far more common than expected. However, episodic memory tests remain an important support method for AD diagnosis, especially in cases with discrepant results between amyloid PET and CSF biomarkers.

## 1. Introduction

Increasingly, middle-aged people suffer from memory loss, subjective complaints without any medical evidence, cognitive impairment derived from other disorders (e.g., anxiety and depression), and dementia, with Alzheimer’s disease (AD) being the most common [1]. AD diagnosis requires a complete clinical evaluation based on neuroimaging, neuropsychological assessment, and amyloid status determination. This status is evaluated by means of biomarkers levels (β-amyloid-42 (Aβ42), total Tau (t-Tau), and phosphorylated Tau (p-Tau)) in cerebrospinal fluid (CSF) or by amyloid positron emission tomography (amyloid PET) [2]; however, these techniques show some discrepant results. Great results consistencies for both diagnostic techniques are described and expected; however, in common specialized practice, this consistency needs to be confirmed.

Regarding the histopathological characteristics of AD, the extracellular formation of amyloid plaques and intracellular formation of neurofibrillary tangles are the main hallmarks; therefore, diagnostic criteria are based on these specific biomarkers. They are classified into amyloid status biomarkers (fibrillary amyloid retention by PET (amyloid PET), and Aβ42, t-Tau, and p-Tau in CSF) and neurodegeneration/topographic biomarkers (temporoparietal metabolism by PET-FDG and medial temporal atrophy by NMR) [3].

The amyloid status biomarkers consist of CSF levels for Aβ42, t-Tau, and p-Tau, which provides relevant information for early AD diagnosis [4]. However, the clinical variability in late-onset AD requires us to consider other biomarkers. In this sense, some trials aim to investigate Aβ42/Aβ40 or APP669-711/Aβ42 ratios [5,6]. Another study suggested that the Aβ42/Aβ40 ratio improved the diagnostic capacity of Aβ42 [7]. Moreover, amyloid PET molecular imaging provides information about the complex interactions between Aβ42, Tau, and neuroinflammation in AD and mild cognitive impairment (MCI), allowing us to differentiate among the changes associated with the normal aging process or the physiopathological processes of the disease. In fact, PET imaging enables preclinical AD detection [8], and it has some advantages, such as being a less invasive technique with high precision and standardization levels. Nevertheless, amyloid PET is more expensive and has some limitations regarding the use of tracers. In fact, tracers are difficult to produce and handle since they have a short half-life. In addition, the different amyloid tracers available in clinical practice could show different information, and their diagnosis indexes (sensitivity, specificity) are not 100% [9].

The recent development of CSF biomarkers and amyloid PET techniques in the early diagnosis of AD demonstrates important advances in the knowledge of the role of the amyloid status [10]. However, some studies from the literature showed discrepant results between CSF analysis and the amyloid PET technique [11,12]. Specifically, atypical CSF patterns showing high levels for p-Tau and/or t-Tau but normal levels for Aβ1-42 are relatively frequent in patients with MCI and AD [13,14].

On the other hand, neuropsychological evaluation tests have shown their utility in the detection of neurodegenerative diseases, such as AD, and could constitute a useful tool to discern doubtful cases [15].

In this sense, the present study is focused on the evaluation of the concordance between the results of CSF biomarkers levels (Aβ1-42, T-tau, and P-tau) and amyloid PET in the diagnosis of patients with cognitive impairment to provide additional tools, such as neuropsychological tests, in order to help in discrepant case diagnosis in clinical practice.

## 2. Materials and Methods

### 2.1. Participants and Samples Collection

The study involved patients from the neurology unit from the Hospital Universitari I Politècnic la Fe (Valencia, Spain) (n = 82), with ages between 48 and 80 years. All of them underwent two procedures for AD diagnosis: (i) lumbar puncture to obtain CSF samples for the determination of AD biomarkers (Amyloid β42 (Aβ42), t-Tau, and p-Tau); and (ii) amyloid PET technique (Florbetapir, Flubetamol). In addition, we evaluated all patients with neuropsychological tests (CDR, RBANS) (see Section 2.2).

We classified participants into two groups: (i) concordant cases (n = 67)—CSF biomarkers and amyloid PET imaging provided the same diagnosis; (ii) discordant cases (n = 15)—CSF biomarkers and amyloid PET imaging provided different diagnoses. We determined CSF positivity by Aβ42 levels with a cut off of 750 pg mL^−1^. The study protocol was approved by the Ethics Committee (CEIC) from the Health Research Institute La Fe (Valencia, Spain). All participants signed the informed consent.

### 2.2. Neuropsychological Evaluation

The neuropsychological evaluation of the participants was based on the CDR and RBANS tests.

The CDR test (clinical dementia rating; Morris, 1993) establishes 5 possible states based on the evaluation of 6 areas of cognitive and behavioral autonomous functioning (memory, orientation, reasoning and problem solving, activities of daily life, domestic tasks and hobbies, and personal care and hygiene). The established states are: 0 (normal cognitive and behavioral functioning), 0.5 (questionable dementia), 1 (mild dementia), 2 (moderate dementia), and 3 (severe dementia) [16].

The RBANS (repeatable battery for neuropsychological status assessment) test is a neuropsychological battery that evaluates 5 areas: immediate memory (IM), language (L), attention (A), delayed memory (DM), and visuospatial construction (VC). A resulting score lower than 85 in some areas would indicate cognitive alteration [15,17].

### 2.3. Statistical Analysis

We summarized participants’ demographic and clinical data using mean (standard deviation) and median (interquartile range) for numerical variables, and absolute frequency (%) for qualitative variables.

We performed an evaluation of the relationship between the results of amyloid PET, biomarkers in CSF (Aβ42, t-Tau, and p-Tau), and neuropsychological evaluation (RBANS and CDR) through logistic regression, including sex and age as covariates. We performed statistical analyses using R software version 4.0 (1) and clickR package version 0.4.39.

## 3. Results

### 3.1. CSF Biomarkers and Amyloid PET Imaging

Table 1 summarizes the demographic and clinical data from the two participant groups. Regarding gender, more discordant and concordant-positive cases were obtained for females compared to males, while concordant-negative cases were mainly males. Additionally, CSF biomarkers (t-Tau, p-Tau, and Aβ42) showed differences between groups. Specifically, as expected, higher levels of Aβ42 and lower levels of t-Tau and p-Tau were found in the negative concordant group compared with the positive concordant group, while medium levels were present in discordant cases. In the same way, neuropsychological scores are, in general, altered in the positive concordant group and are normal in the negative concordant group, while the discordant group showed values closer to the cut-off values.

**Figure 1:**
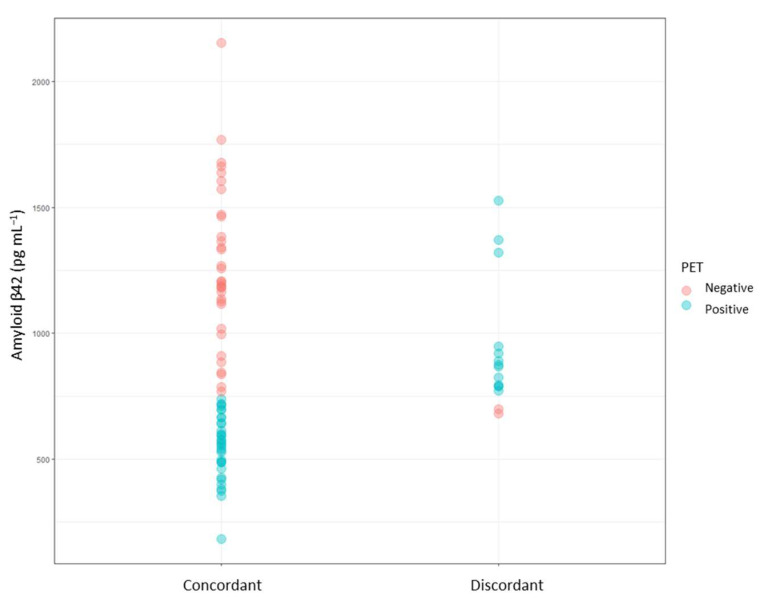
Representation of CSF biomarker level (Aβ1-42) and amyloid PET results for both participant groups (concordant, discordant).

**Figure 2:**
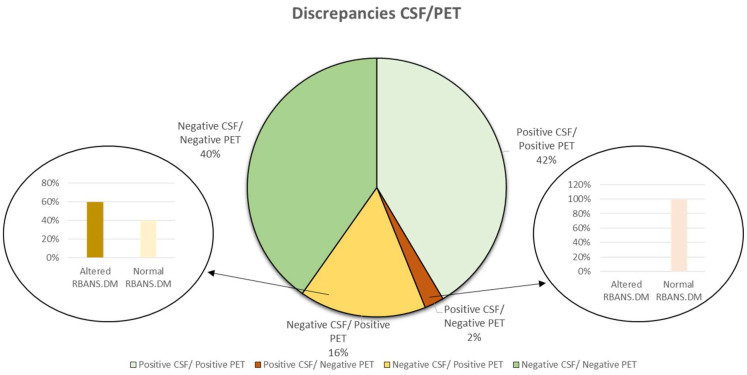
Representation of concordant and discordant results among CSF biomarkers and amyloid PET.

Figure 1 shows the levels of the CSF biomarker (Aβ42) and the amyloid PET results in both participant groups, showing that around 18% of cases were discrepant. Additionally, it can be seen that discrepant results are mainly from intermediate CSF levels, which are close to the cut-off point (750 pg mL^−1^). In addition, Figure 2 shows that most of the discrepant cases correspond to patients with positive amyloid PET (1) and negative CSF biomarkers (Aβ42 levels ≥ 750 pg mL^−1^). Similarly, a comparison between amyloid PET and the ratio between t-Tau and Aβ42 showed 18% of discrepant cases.

### 3.2. Biomarkers and Neuropsychological Evaluation

The diagnosis potential of the neuropsychological evaluation was evaluated with special attention to discordant cases; specifically, the scores obtained from CDR and RBANS. DM tests were assessed as complementary tools to the biochemical and imaging tests. For discrepant cases, patients with negative CSF biomarkers and positive PET manifest alterations in episodic memory in 60% of cases (see Figure 2). By contrast, all the cases (only two patients) with positive CSF biomarkers and negative PET showed normal episodic memory performance.

**Figure 3:**
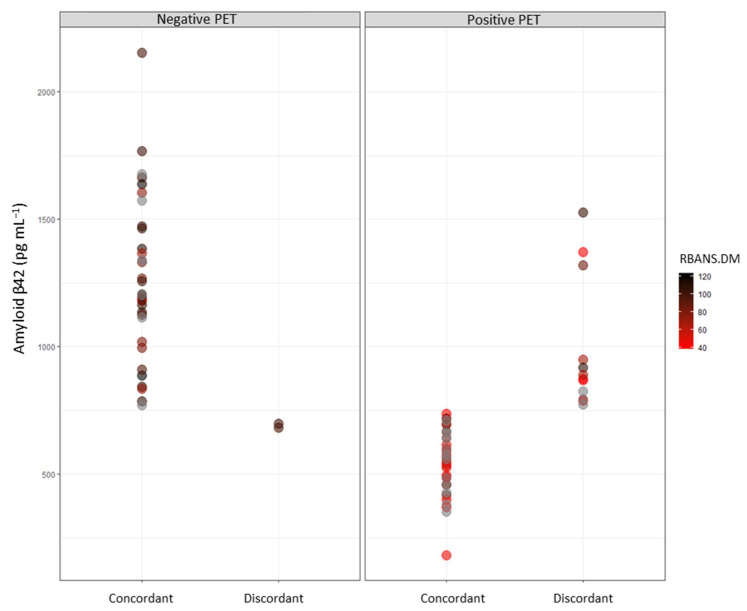
Relationship between Aβ1-42 CSF and RBANS.DM levels depending on the amyloid PET result; negative PET on the left, positive PET on the right.

The relationship between Aβ42 levels and CSF and RBANS.DM tests is shown in Figure 3. As observed, PET-negative amyloids are shown on the left side, with PET-positive amyloids on the right. Among negative PET cases, concordant cases showed Aβ1-42 levels > 750 pg mL^−1^, and most of them had high RBANS.DM scores (dark values); however, discordant cases showed Aβ42 levels < 750 pg mL^−1^ and high RBANS.DM scores. Among positive PET cases, concordant cases showed Aβ42 levels < 750 pg mL^−1^, and most of them showed low RBANS.DM scores; furthermore, discordant cases showed Aβ42 levels > 750 pg mL^−1^ and low RBANS.DM scores.

**Figure 4:**
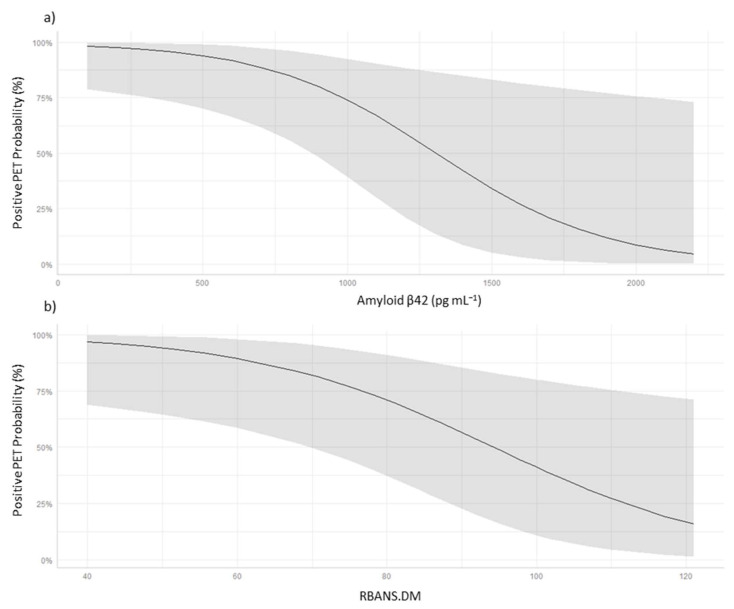
(**a**) Conditional effects of Aβ1-42 levels in CSF over the probability of positive amyloid PET; (**b**) conditional effects of RBANS.DM scores over the probability of positive amyloid PET.

Figure 4 depicts linear regression, and we can see the conditional effects of the variables between the Aβ42 levels and RBANS.DM scores on the probability of amyloid PET positivity. The line shows the probability of positive PET for each Aβ42 level (Figure 4a) or RBANS.DM score (Figure 4b), and the shaded part represents the confidence interval. As can be seen, the confidence intervals are wide at high Aβ42 levels and RBANS.DM scores, which could reflect considerable variability in the classification of patients according to amyloid PET results, especially in cases with high levels of Aβ42 in CSF and high scores in RBANS.DM. Among the CDR results, there was no correlation between CSF biomarkers levels in the same way that happens in the other RBANS subsets.

**Figure 5:**
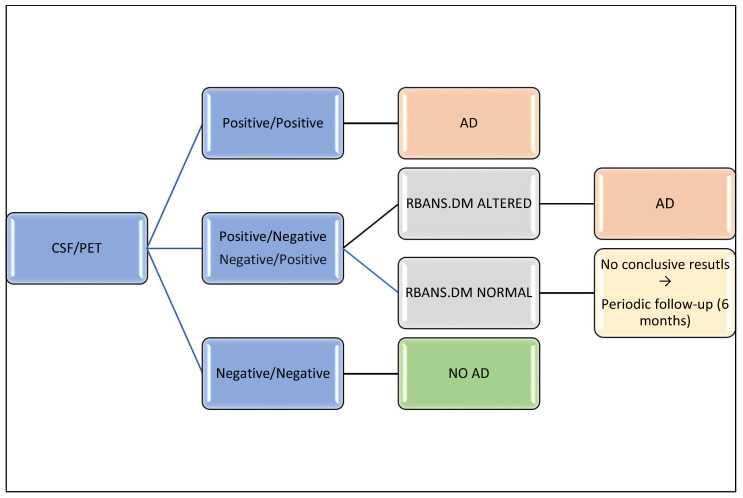
Decision tree for AD diagnosis based on CSF biomarkers, amyloid PET, and RBANS.DM score.

Figure 5 shows a decision tree for AD diagnosis based on these results. As can be seen, patients with positivity and negativity for both tests should be diagnosed with AD and non-AD, respectively. In the discrepant cases, RBANS.DM could be used to clarify the diagnosis. In cases with altered RBANS.DM, patients should be diagnosed with AD, while patients with normal scores for RBANS.DM should be should be followed up to define their diagnosis.

## 4. Discussion

At present, the gold standard for the diagnosis of AD is based on the evaluation of the amyloid status by means of CSF biomarker Aβ42 or amyloid PET imaging [11]. In this work, an assessment of the concordance between the results from CSF biomarker levels (Aβ42) and amyloid PET tests was carried out in order to compare their reliability in the diagnosis of patients with cognitive impairment in a real clinical practice context.

Sometimes test results are inconclusive or lack agreement. Moreover, some studies showed discrepancies between both techniques [11,12]. Additionally, previous studies indicated that amyloid PET and CSF biomarkers might not reflect identical clinical information; therefore, a combination of both techniques could be the best option to characterize clinically unclear cognitive impairment [18]. In the present work, most of the discrepant cases were patients with negative CSF biomarkers and positive PET results. These results are contrary to those described by Hye et al., who concluded that CSF biomarkers were more sensitive than PET for AD diagnosis [19]. In addition, a previous study showed similar discordant results between Aβ42 and amyloid PET, with 25% discrepancies [20]. In general, this is a very high discrepancy level for two analytical techniques considered the gold standard in AD diagnosis. Regarding the amyloid deposition, previous studies described the deposition of peptides Aβ37 or Aβ39 in extracellular plaques [21]. In addition, perivascular deposits in some amyloid plaques could contribute to high variability in amyloid PET results. Therefore, CSF Aβ42 and amyloid PET may not exactly reflect the same information. Additionally, a previous study found that the Aβ42/Aβ40 ratio showed better concordance with amyloid PET results [22]. However, the t-Tau/Aβ42 ratio showed similar results to Aβ42 in our study. These differences among studies could be explained by the different cut-off points used, as well as by the differences in the selection of participants. In the present work, the participants were patients from the Cognitive Disorders Unit in the Hospital La Fe, implying some selection bias. Regarding the clinical implications of the discrepant results, it is important to highlight that both tests (CSF Aβ42 and amyloid PET) were only applied to cases showing some inconsistent results between a diagnosis test and the clinical manifestations. Similarly, previous studies showed that both techniques were applied when there was a mismatch between the clinical diagnosis and the biomarker result [23]; furthermore, a study by Wilde et al. showed that discordant results provided important information about clinical progression [24]. In this sense, any discordant negative result (Aβ42 CSF or amyloid PET) should be validated by means of the other test.

According to gender differences, we found higher discrepancies and higher positive results in females. The higher proportion of positive females could be explained by their higher risk levels for AD [25], as well as the higher number of female participants in this study. In addition, these could be explained by the influence of other factors, such as the ApoE genotype (not available in our data) [26]. Therefore, studies in other cohorts are necessary to confirm these results.

Regarding neuropsychology tests, they are mainly used to detect cognitive impairment cases and to evaluate disease progression [27]. In this sense, the CDR test is the most used classification system in clinical dementia research [28]. It consists of a global clinical scale, which measures social, behavioral, and functional changes in patients. Among its advantages is that it is independent of other psychometric tests, it does not require a baseline evaluation, and it can be used as a control for each individual [29]. On the other hand, RBANS is a short neuropsychological battery with high sensitivity for the detection of cognitive disorders in degenerative and non-degenerative pathologies. It has been adapted into several languages and is widely used in some countries [30]. RBANS scores produce excellent estimates of diagnosis accuracy and it constitutes a useful tool in the detection of cognitive deficits associated with AD [17,31,32]. Specifically, the RBANS.DM domain could be a cost-effective tool for identifying the early signs of AD pathology, improving clinical decisions about the progression to dementia due to AD [15].

In the present study, the agreement between a CSF biomarker (Aβ42), and the amyloid PET technique was evaluated. The results showed that those subjects with alterations in amyloid PET (positive) could be AD patients; however, if CSF Aβ42 levels are ≥the cut-off point (negative), the AD diagnosis was not corroborated. In these discrepant cases, the patients’ cognitive impairment was evaluated according to the RBANS.DM domain, which constitutes an important tool in establishing diagnosis, much as it is in cases without studying amyloid status.

An episodic memory assessment (RBANS.DM) would allow us to establish the phase of the disease (the lower score obtained in this area, the greater memory deficit), as the study by Hammers et al. already pointed out. In fact, they claimed that RBANS.DM could illuminate clinical decision making regarding the possible progression to dementia due to AD [27]. In addition, evaluations of episodic memory with tests such as the RBANS.DM scale would remain a key tool to identify patients with AD, even when specific amyloid detection techniques are fully available. So, its use is quite able to reduce economic costs in those countries where amyloid PET cannot be performed by public health systems. Similar results were obtained from a previous study [27]. In the present study, RBANS.DM showed a great capacity to predict amyloid PET status. For that reason, RBANS.DM could be employed as an AD diagnosis approach in cases where economic conditions do not allow the performance of more expensive tests, such as amyloid PET. In addition, episodic memory tests (e.g., RBANS.DM) could be useful as a screening test for AD diagnosis due to its high sensitivity. However, its application requires further resources (specialized staff and time), which are not available in most health systems.

The main limitations of this study are the small sample size and the reduced number of discordant cases. In addition, the participants were patients from the Neurology Unit; therefore, the sample could be biased. Moreover, the specific cut-off point used according to clinical practice in this unit could be different from others, making it difficult to extrapolate the results. Regarding amyloid PET, the interpretation of these results can be more subjective, especially in the most borderline cases. So, highly specialized personnel are required to be in charge of these tasks. Similarly, for neuropsychological evaluation, some specialized staff is required.

## 5. Conclusions

Alzheimer’s disease diagnosis is mainly based on amyloid status (Aβ42 in CSF and amyloid PET). However, in some cases, both techniques presented discordant results. In these cases, classical complementary non-invasive and cost-effective neuropsychological tools continued providing the key data to support AD diagnosis. In this sense, episodic memory assessments still constitute a useful tool in supporting the diagnosis of patients with risks for Alzheimer’s disease development. Therefore, neuropsychological evaluations could help to increase knowledge regarding the patient’s cognitive impairment degree or disease progression but also identify AD patients.

In general, this study highlights the high percentage of discrepancies between two techniques considered the gold standard in the diagnosis of AD. In this sense, there is an increasing need to carry out further research in plasma samples to identify new specific biomarkers that are minimally invasive and economically affordable.

## Figures and Tables

**Table 1:** Demographic and clinical characteristics of participants.

Variable		Concordant Cases (n = 67)		Discordant Cases (+,−) (−,+) (n = 15)
		Positive (+,+) (n = 34)	Negative (−,−) (n = 33)	
Age (years, median (IQR))		71 (64.75, 74)	66 (63, 71.5)	69 (65.5, 75)
Sex (female, n (%))		20 (58.82%)	8 (24.24%)	10 (66.67%)
Aβ1-42 (pg mL^−1^, median (IQR))		570 (486, 665.25)	1204 (1067.25, 1468)	868 (789.5, 933.5)
t-Tau (pg mL^−1^, median (IQR))		513 (335.5, 619.5)	249 (142, 407.5)	438 (346.5, 641.5)
t-Tau/Aβ1-42		0.825 (0.59, 1.34)	0.2 (0.14, 0.27)	0.52 (0.3, 0.76)
p-Tau (pg mL^−1^, median (IQR))		74 (55.5, 97.75)	49 (38, 72)	77 (59, 83.5)
RBANS.IM (score, median (IQR))		69 (53, 81)	87 (76, 100)	82 (77.5, 86.25)
RBANS.VC (score, median (IQR))		81 (78, 92)	96 (81, 112)	88 (84, 100.5)
RBANS.L (score, median (IQR))		85 (78, 88)	92 (78, 96)	75.5 (60, 82)
RBANS.A (score, median (IQR))		72 (60, 82)	94 (79, 112)	80.5 (74.25, 91.75)
RBANS.DM (score, median (IQR))		48 (40, 60)	98 (81, 103)	68 (40, 98.75)
CDR (n, (%))	0	3 (13.04%)	21 (77.77%)	5 (41.67%)
	0.5	16 (69.56%)	5 (15.52%)	7 (58.33%)
	1	4 (17.39%)	1 (3.70%)	0 (0%)

IM: immediate memory, L: language, A: attention, DM: delayed memory, VC: visuospatial construction.

## Data Availability

The data presented in this study are available on request from the corresponding author.
